# Robust Tracking and Quantification of Open-Beak Behavior for Heat Stress Assessment in Multi-Tier Caged Rearing Broiler Breeders via Mobile Inspection

**DOI:** 10.3390/ani16152408

**Published:** 2026-08-04

**Authors:** Ning Kong, Tongshuai Liu, Guoming Li, Lei Xi, Shuo Wang, Yuepeng Shi

**Affiliations:** 1 School of Energy and Intelligence Engineering, Henan University of Animal Husbandry and Economy, Zhengzhou 450044, China; ningkong@hnuahe.edu.cn (N.K.); 80772@hnuahe.edu.cn (Y.S.); 2 College of Animal Science & Technology, Henan University of Animal Husbandry and Economy, Zhengzhou 450046, China; 3 Henan Engineering Research Center on Animal Healthy Environment and Intelligent Equipment, Zhengzhou 450046, China; 4 Department of Poultry Science, The University of Georgia, Athens, GA 30602, USA; gmli@uga.edu; 5 Institute for Artificial Intelligence, The University of Georgia, Athens, GA 30602, USA; 6 School of Information Engineering, Henan University of Animal Husbandry and Economy, Zhengzhou 450046, China; 81239@hnuahe.edu.cn

**Keywords:** animal welfare monitoring, heat stress assessment, multi-object tracking, cumulative panting ratio, trajectory stitcher

## Abstract

Panting is one of the earliest and most prominent behavioral responses of chickens under heat stress. More importantly, panting is inherently a temporally continuous state rather than an instantaneous event. However, existing vision-based methods usually determine panting from beak opening in individual frames, which contradicts the nature of panting and therefore cannot provide reliable assessment. To address this limitation, this study proposes a Cumulative Panting Ratio (CPR), which quantifies sustained open-beak behavior associated with panting by tracking and accumulating instantaneous open-beak events within a temporal sliding window. To support consistent estimation of sustained open-beak behavior and CPR, a cascaded Detection–Tracking–Classification pipeline is constructed to explicitly decouple body-level tracking from fine-grained beak behavior inference, thereby reducing the conflict between global localization and local feature extraction. In addition, a Near-Online Trajectory Stitcher is proposed to reconnect fragmented tracklets using unified temporal, spatial, scale, and directional constraints induced by platform movement. Experiments on practical inspection datasets demonstrated robust tracking continuity and stable quantification performance. Compared with state-of-the-art frameworks, the proposed method reduced identity switches from 10 to 4 and improved IDF1 by 2.43%, while minimizing counting errors across diverse detector architectures. With TensorRT acceleration and multi-stream parallel processing on the Jetson Orin NX, system throughput increased from 7.89 FPS to 51.43 FPS without compromising accuracy. Overall, this work provides a practical solution for quantitative heat stress assessment and supports the deployment of physiological state monitoring systems for precision livestock farming.

## Simple Summary

Broiler breeders in multi-tier cage systems are vulnerable to heat stress, particularly in summer. Panting, characterized by sustained open-beak behavior accompanied by an accelerated respiration rate, is an early indicator of thermal discomfort. Timely detection enables ventilation and cooling adjustments before heat exposure compromises bird health and productivity. Manual flock inspection is labor-intensive and inconsistent, whereas image-based methods relying on isolated frames may misclassify sustained open-beak behavior that develops over time. We developed a mobile inspection system in which trolley-mounted cameras continuously track individual birds along cage rows. The system quantifies sustained open-beak behavior by aggregating open-beak detections for each bird over a one-second window. A trajectory-repair mechanism preserves identity during temporary occlusion by cage structures. Commercial farm evaluation demonstrated that the system could perform near-online behavioral quantification on a compact edge computing device, with the obtained measurements showing associations with environmental heat load. The proposed approach provides a practical tool for automated monitoring of heat stress-related behavioral responses and may support animal welfare management in precision poultry production.

## 1. Introduction

The wave of precision livestock farming is rapidly transforming China’s animal husbandry industry, particularly the poultry sector, shifting management paradigms from coarse group-level supervision to fine-grained individual monitoring [1,2,3]. For broiler breeders, which possess relatively high individual economic value, precise management during growth is especially critical. The rearing period represents a pivotal transitional stage in the life cycle of broiler breeders, directly influencing their body development and subsequent reproductive performance [4]. Currently, high-density multi-tier cage systems are the predominant rearing mode for broiler breeders in China. Under such conditions, prolonged heat stress may induce severe physiological and metabolic disorders, causing irreversible damage to breeder survival rates and egg quality [5]. Timely detection of heat stress and appropriate environmental interventions are therefore essential to ensure flock developmental quality and production potential, enhance animal welfare, and ultimately improve the economic efficiency of poultry farming operations.

However, in commercial poultry farms, the timely detection and accurate assessment of heat stress remain highly challenging. On the one hand, traditional manual inspection is labor-intensive, inefficient, and inherently subjective. On the other hand, although computer vision-based intelligent inspection systems can effectively address the above limitations, their practical deployment is still constrained by harsh physical conditions. During the rearing period, to control sexual maturity and suppress injurious pecking behavior, modern broiler breeder rearing houses are typically maintained under very low illumination levels. Meanwhile, the vertical bar structures of cages introduce severe occlusions that fragment the camera’s field of view under mobile inspection. In video streams acquired by mobile inspection cameras, insufficient lighting combined with frequent inter-individual occlusions often leads to large numbers of false negatives and false positives in object detection and behavior classification. Moreover, the homogeneous appearance of broiler breeders and the ego-motion of the mobile inspection perspective further aggravate trajectory fragmentation and frequent identity switching in multi-object tracking (MOT). Such perception errors at the visual sensing stage do not remain isolated, instead, they propagate through the processing pipeline and ultimately amplify into systematic statistical biases in downstream analytical results.

Conventional heat stress assessment has primarily relied on environmental indices such as the Temperature–Humidity Index (THI) [6]. However, these physical parameters are inherently limited and often fail to accurately reflect the actual physiological heat load experienced by individual birds. In contrast, behavioral responses of broilers, as direct and observable manifestations of their adaptation to heat stress, provide a more direct and sensitive indication of the birds’ actual thermal state [7,8].

Accordingly, researchers have investigated birds’ behavioral responses under controlled temperature and humidity conditions to identify observable indicators associated with heat stress. Oso et al. [9] proposed the Bird Activity Index, which quantifies inter-frame pixel differences in motion sequences and clusters them to characterize activity levels. When interpreted alongside environmental conditions, the index showed a strong association with heat stress. Zhou et al. [10] developed a behavior–environment association model for laying hens and identified six representative behaviors across different levels of heat stress. Among these behaviors, panting has been recognized as one of the most direct and prominent indicators, and their YOLO-SPS model achieved a detection mAP of 97.5%. Collectively, these studies support a transition from environment-based assessment toward animal-centered heat stress monitoring.

Building on this behavioral paradigm, researchers have explored computer vision methods to automatically recognize heat stress-related behaviors, progressing from conventional machine learning techniques to modern deep learning-based approaches. Early work by Pereira et al. [11] employed image processing combined with decision tree algorithms to recognize behaviors of white broiler breeder hens, achieving an accuracy of 70.3% under experimental conditions. Yang et al. [12] integrated YOLOv8 for behavior detection with DeepSORT for MOT to identify piling and smothering behaviors in cage-free hens, while also quantifying overall flock activity levels, achieving a MOTA of 94% and an IDF1 of 90%. Wang et al. [13] proposed a two-stage binary classification framework combining an improved YOLOv8n with MobileNetV3, utilizing color features of the comb, beak, and eyes to identify low-yield hens, with an accuracy of 97.65%. Hu et al. [14] introduced a CBLFormer model for behavior recognition and temporal localization, capable of precisely identifying transition points and durations of behaviors, achieving an average accuracy of 96.93% across six common behaviors in real farming environments. Li et al. [15] developed an improved YOLOv8-based model for abnormal beak detection in caged layer chickens, reaching a detection accuracy of 92.7%. Cardoen et al. [16] proposed a multi-camera view fusion pipeline for broiler localization and tracking, incorporating an unsupervised tracking method named tracking-by-curve-matching to enhance robustness, achieving a MOTA of 81% and an IDF1 of 89%. Yu et al. [17] presented a 3D behavior recognition framework called CSP-YOWO-TrajNet that integrates spatiotemporal information for detecting heat stress-related behaviors in laying hens, achieving a MOTA of 89.2% and an IDF1 of 95.4%. Overall, these studies have enabled qualitative recognition of individual or flock-level behaviors; however, they lack robust quantitative metrics, making it difficult to infer the severity of underlying heat stress and, consequently, constraining the ability to support precise environmental control.

Beyond behavioral analysis, physiological and phenotypic indicators such as body temperature, body weight and morphological traits have also been explored to provide deeper insight into the actual physiological heat load experienced by individual birds. Kou et al. [18] proposed a novel method integrating CT imaging with a meta-learning segmentation network for non-invasive, in vivo measurement of testicular volume and weight in broiler breeders, achieving an mDSC of 0.63 and a volume–weight correlation coefficient of 66.18%. Zheng et al. [19] developed a live body weight prediction approach based on single-view point cloud data, using an improved PointNet++ model to regress weight from top-view back surface scans, achieving a mean absolute error (MAE) of 95 g and a relative error of 6.66%, with a Pearson correlation coefficient of 0.8817 on Huainan and Huxu mixed-breed datasets. Wang et al. [20] proposed a lightweight model named YOLOv8n-mvc that integrates MobileViT and coordinate attention, combining RGB, thermal infrared, and depth data to enable precise chicken head localization and real-time temperature measurement in practical farming environments. Yang et al. [21] proposed a Transformer-based multimodal detection framework that integrates visible and thermal infrared imagery with an improved RT-DETR model. This approach achieved mAP@0.5 scores of 0.968 and 0.951 for detecting dead and abnormal hens, respectively. Despite the deeper analyses, these approaches face two major limitations: first, the high computational complexity and operational cost hinder deployment on mobile inspection platforms; second, they are highly sensitive to environmental variations such as illumination, temperature, humidity, and camera-to-object distance, limiting their generalizability.

In summary, existing research has primarily focused on cage-free or floor-rearing systems, with relatively few addressing actual cage-rearing scenarios. Most focus on laying hens or broilers, while broiler breeders remain comparatively understudied; fixed-viewpoint monitoring scenarios are far more common than mobile inspection scenarios; and behavior recognition based on instantaneous single-frame images predominates, whereas studies incorporating both spatial and temporal domains remain limited [22,23,24,25,26]. Compared with these relatively open or well-controlled environments, multi-tier caged broiler breeder houses during the rearing period present significantly greater challenges. Under conditions of low illumination, occlusion caused by cage structures, and dynamic ego-motion from mobile inspection, errors in object detection and MOT are more prone to accumulate, ultimately degrading the accuracy of heat stress quantification. Therefore, there is an urgent need for robust methods customized to mobile inspection scenarios.

**Figure 1:**
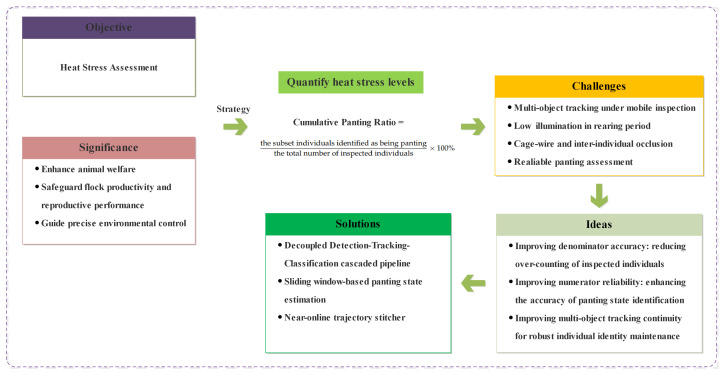
Research map of this study.

Figure 1 presents the research map of this study, illustrating the logical progression from the research objective and significance through key challenges and methodological ideas to the proposed solutions. To address the aforementioned limitations, this study proposes the following contributions:

1. A novel statistical indicator is introduced based on individual behavior recognition and MOT under mobile inspection scenarios. By continuously counting the total number of inspected birds and the subset exhibiting sustained open-beak behavior, the proposed method estimates the Cumulative Panting Ratio (CPR) at the flock level. This enables a shift from single-frame instantaneous detection to spatiotemporally continuous quantification of sustained open-beak behavior associated with heat stress.
2. To improve tracking stability under mobile inspection conditions, a cascaded Detection–Tracking–Classification framework is further developed to decouple global body localization from fine-grained open-beak recognition. By separating these two tasks, the proposed framework reduces the feature conflict between large-scale target tracking and subtle beak behavior classification, thereby enabling more stable downstream trajectory association.
3. To reduce counting errors induced by severe occlusion and feature degradation in low-light cage environments, a detector-agnostic post-processing strategy termed Near-Online Trajectory Stitcher is proposed. By incorporating the directional motion prior of the mobile inspection platform, the proposed strategy reliably associates and reconstructs fragmented trajectories, thereby improving counting accuracy during mobile inspection.
4. A parallelized perception architecture is constructed for multi-tier cage environments. By synchronously processing multiple video streams and leveraging TensorRT acceleration, the system achieves real-time, multi-tier coverage monitoring on the NVIDIA Jetson Orin NX platform.

The remainder of this paper is organized as follows. Section 2 describes the data acquisition process and dataset preparation procedures. Section 3 details the proposed framework and its main components. Section 4 presents the experimental evaluation of the proposed methods. Section 5 interprets the findings and discusses their implications and limitations. Finally, Section 6 summarizes the main conclusions and outlines directions for future research.

## 2. Materials

**Figure 2:**
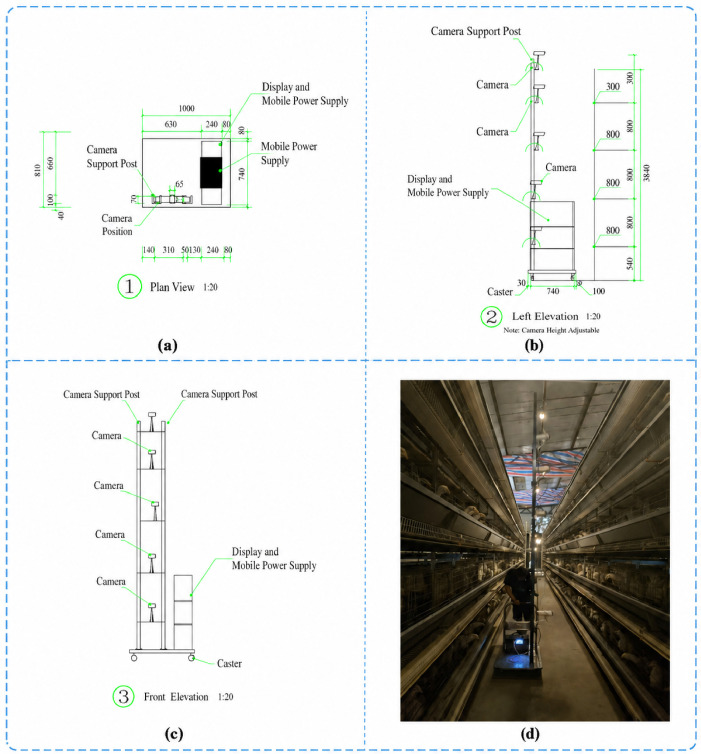
Design and deployment of the mobile data acquisition platform: (**a**) planar structural design of the platform; (**b**) left-side elevation CAD schematic; (**c**) front elevation CAD schematic; and (**d**) practical operation scene of the platform.

To obtain activity videos of broiler breeders under commercial feeding conditions, a mobile data acquisition platform was constructed in this study (Figure 2). The system was equipped with three EZVIZ CS-C5S-3C2WFR cameras (Hangzhou EZVIZ Network Co., Ltd., Hangzhou, China) featuring infrared night vision to adapt to the low-light conditions inside the poultry house. The cameras were horizontally oriented to align with each layer of birds and captured videos at a resolution of 1920 × 1080 pixels and a frame rate of 25 FPS. The recorded videos were stored locally onto an internal 128 GB MicroSD card within each camera and exported at regular intervals. Data were collected at Hongfeng Poultry Co., Ltd., in Yancheng District, Luohe City, Henan Province, China, from July to September 2025. To capture variations in environmental and lighting conditions while avoiding disruption to routine farm operations, recordings were conducted at three representative time points (08:00, 15:00, and 20:00) on each sampling day. The subjects were AA+ broiler breeder pullets (Arbor Acres, Aviagen) aged 14–21 weeks. No experimental intervention was imposed on the birds during data collection.

The platform was designed to simulate the working perspective and motion trajectory of a future mobile inspection robot. During data collection, the platform was manually pushed along the central aisle at an approximately constant speed of 0.35 m/s, ensuring smooth scanning of each cage tier while minimizing disturbances caused by chassis vibration. This setup was intended to produce data representative of mobile inspection under commercial production conditions for evaluating the proposed method.

Subsequently, the collected data were manually screened, segmented, and annotated under the guidance of poultry-production experts to construct three datasets: D1, D2, and D3. The annotation process was conducted using X-AnyLabeling (version 3.2.2) [27], which supports model-assisted automatic labeling. Specifically, a small subset of data was manually annotated in the initial stage to train a preliminary detection model. This model was then employed to generate coarse annotations for the remaining data, followed by manual refinement to ensure annotation accuracy. This human-in-the-loop strategy significantly improved labeling efficiency while maintaining high-quality annotations.

**Figure 3:**
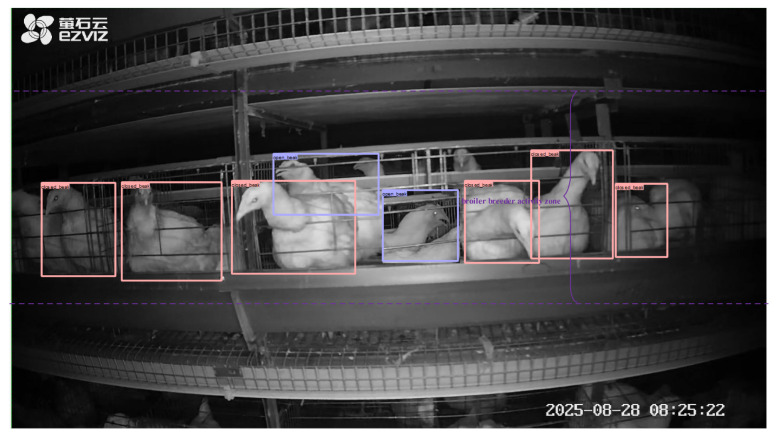
Annotation example of the constructed dataset D1. The soft pink bounding box is the “closed_beak” class, the soft blue bounding box is the “open_beak” class, and the purple bracketed region in the center denotes the broiler breeder activity zone.

The first dataset, D1, was constructed to train the object detector and beak behavior classifier. From the videos recorded on four selected days, 10,089 images were extracted. Among these images, 1260 images from independent video sequences were reserved for validation, while the remaining images were considered training candidates. Birds were annotated into two categories based on beak behavior, namely “closed_beak” and “open_beak”, with bounding boxes covering the visible body regions. Under typical behavioral conditions of the flock, the proportion of birds exhibiting open-beak behavior is inherently low. Moreover, even for those birds, the occurrence of this behavior occupies only a small fraction of their daily activity. This observation is further confirmed by the collected dataset. Statistical analysis shows that the ratio of closed-beak to open-beak instances exceeds 24:1 on average, leading to a severe class imbalance that adversely affects detection performance. To mitigate this issue, a two-stage data augmentation strategy was adopted. First, 146 cropped samples of open-beak birds were manually extracted from the recorded videos. Then, 2000 images were selected from the original training candidates and augmented using an off-line strategy termed Constrained Copy–Paste. Due to continuous video recording, the original training candidates contained substantial temporal redundancy, with many adjacent frames exhibiting highly similar bird poses and background conditions. Therefore, instead of using all extracted frames, a representative subset of 2000 images was selected to reduce redundant samples while maintaining diversity in bird appearance, occlusion patterns, and behavioral states. The remaining extracted frames were not used for model training or validation. The “constrained” term refers to the incorporation of spatial priors, restricting the placement of pasted samples to realistic "broiler activity zones", as shown in Figure 3. This procedure increased the number of open-beak instances while maintaining the original number of training images. After augmentation, the final D1 dataset consisted of 3260 images, including 2000 training images processed by Constrained Copy–Paste and 1260 untouched validation images. A re-statistical analysis shows that, within the 2000 augmented training images, there are 11,124 closed-beak instances and 3153 open-beak instances, significantly reducing the learning difficulty for the model. The validation set remains unaltered, containing 6825 closed-beak and 305 open-beak instances.

**Figure 4:**
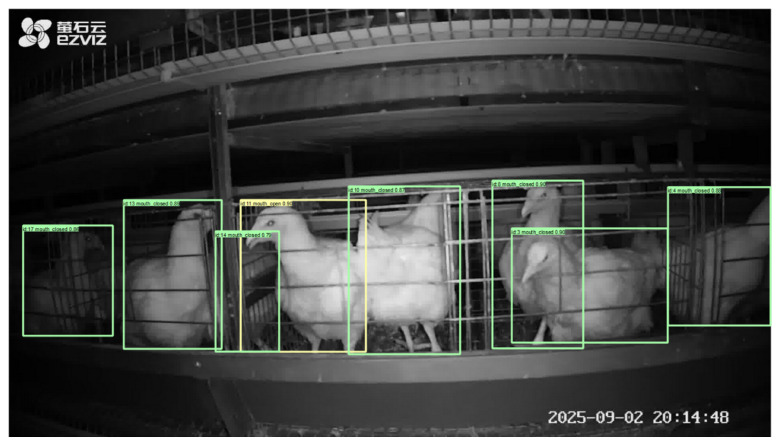
Annotation frame sample of the constructed dataset D2.

The second dataset, D2, was constructed to evaluate system performance in both tracking and sustained open-beak behavior quantification, which contained temporally continuous ground truth for both identity labels and behavior classes. The recorded footage was first manually reviewed, from which three clear and smooth video clips—each lasting 1 min—were selected at three representative time points (08:00, 15:00, and 20:00) across different days. The original videos were then uniformly downsampled to 10 FPS before frame extraction and manual annotation. Unlike image-level detection benchmarks where samples are independent, MOT-based evaluation [28,29] requires temporally consistent identity labeling across all sequential frames. For a 1 min video at 10 FPS, annotating 600 frames with strict ID consistency and individual beak behavior classes involves greater annotation complexity than handling independent images. Ultimately, a temporally continuous test dataset comprising 1800 images was established. An annotation sample is shown in Figure 4.

While D2 offers precise frame-level ground truth, its 1 min sequences cannot fully capture long-term CPR dynamics. Therefore, a single 6 min video sequence (D3, 10FPS, 3602 frames) was collected, covering a complete mobile inspection from one end of a cage aisle to the other. To ensure independence, D3 was selected on a different recording date with birds of a different age from D1 and D2. Specifically, an expert manually recorded ground-truth bird counts and panting behaviors at 30 s intervals, generating 13 paired CPR observation points. This sampling protocol aligns with our cumulative CPR estimation strategy, allowing for realistic validation of the system performance.

The fourth dataset, D4, was constructed to explore the association between CPR and environmental heat load under commercial farming conditions, using the same broiler breeder house as D1–D3. The house comprised four rows with five vertical tiers. Considering the symmetric layout, one side of the house (two rows) was selected for spatial sampling. Within each row, three longitudinal positions (front, middle, and rear) and three vertical tiers (the bottom two and the top one) were designated, yielding 18 predefined observation zones, each corresponding to a stable cohort of 50 birds in adjacent cages. These zones were repeatedly monitored at regular intervals throughout the production period, ultimately yielding 1097 observation records covering bird ages from 103 to 147 days. For each record, bird age, local ambient temperature (°C), relative humidity (%), and wind speed (m/s) were logged concurrently with a manual count of panting individuals; dividing this count by the fixed zone population of 50 yielded the corresponding CPR at a resolution of 2%. Given the repeated-measures structure of the data, D4 is treated as exploratory rather than confirmatory. The analysis aims to investigate the association between CPR and environmental heat load, without inferring causal relationships.

To provide a clear overview, the key characteristics of all four datasets are summarized in Table 1. Notably, the dataset partitioning was performed at the video-sequence level. Since the inspection camera automatically segmented continuous recordings into individual video files during data storage, each video file was treated as an independent recording unit. For D1, video-level partitioning was conducted before frame extraction and augmentation to ensure that images originating from the same recording unit were not distributed across different subsets. Frames extracted from the same video file were assigned exclusively to a single subset, and no video segment was shared across training, validation, or evaluation subsets.

## 3. Methodology

**Figure 5:**
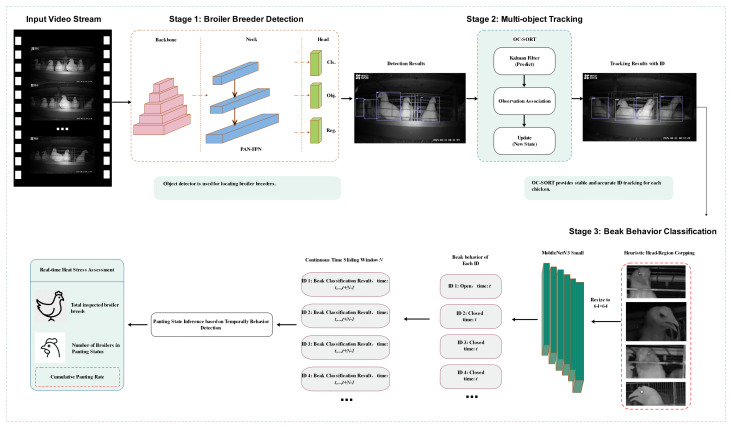
The proposed method for flock-level quantification of sustained open-beak behavior associated with heat stress during mobile inspection.

In this study, we propose a near-online framework for quantifying sustained open-beak behavior associated with heat stress in multi-tier broiler breeder houses, supporting spatially distributed and temporally accumulated flock-level behavioral assessment during mobile inspection. As illustrated in Figure 5, the framework is designed for mobile inspection under low-light, high-density, and severe-occlusion conditions and comprises four main components: (1) a Cumulative Panting Ratio (CPR) for quantifying sustained open-beak behavior based on individual behavior recognition and MOT; (2) a cascaded Detection–Tracking–Classification framework that decouples global bird localization from fine-grained open-beak recognition, enabling more reliable quantification of sustained open-beak behavior through temporal aggregation; (3) a detector-agnostic near-online trajectory stitching method for reconstructing fragmented trajectories; and (4) a parallel perception architecture that enables real-time, multi-tier inspection on edge devices. The following subsections describe these components in detail.

### 3.1. Cumulative Panting Ratio Indicator

Existing vision-based methods typically infer panting from independent frames, which may confuse transient beak-opening events or short-term classification errors with sustained panting and may repeatedly count the same bird during mobile inspection. To address these limitations, we first estimate the sustained open-beak behavior associated with panting of each tracked individual by aggregating open-beak probabilities over a temporal window, thereby improving the robustness of behavioral quantification. Based on the accumulated open-beak behavior of non-duplicate individuals, we then introduce the CPR as an automated measure of flock-level sustained open-beak behavior prevalence, which can serve as a behavioral indicator associated with heat stress.

Let the inspection start time be t=0 and the current time be *t*. $S_{k}\left(t\right)$ denotes the panting state of the bird assigned identity *k* at time *t*. For each tracked bird, the open-beak probability sequence within a sliding window of *N* frames is defined as $P_{N\_frames}=\left\{P_{k,1},P_{k,2},\ldots ,P_{k,N}\right\}$. Its mean $mean(P_{N\_frames})$ and standard deviation $\sigma (P_{N\_frames})$ are used to distinguish sustained open-beak activity from transient events and unstable predictions. Given the adjustable threshold parameters τ and $\sigma_{max}$, $S_{k}\left(t\right)$ is determined as follows:

(1) $$S_{k}\left(t\right)=\left\{\begin{array}{cc} 1, & \mathrm{mean}\left(P_{N\_frames}\right)>\tau ,\sigma \left(P_{N\_frames}\right)\in \left[0,\sigma_{max}\right]\\ 0, & \mathrm{otherwise} \end{array}\right.$$

Based on this, the Cumulative Panting Ratio is defined as follows:

(2) $$CPR\left(t\right)=\frac{\sum_{k\in J_{valid}\left(t\right)}S_{k}\left(t\right)}{|J_{valid}\left(t\right)|}\times 100\%$$

where $J_{valid}\left(t\right)$ represents the total number of validly inspected individuals at the current moment, and $\sum_{k\in J_{valid}\left(t\right)}S_{k}\left(t\right)$ denotes the number of unique individuals exhibiting sustained open-beak behavior up to the current time. Note that $S_{k}\left(t\right)$ represents the cumulative sustained open-beak status of individual *k* up to time *t*: once a bird satisfies the sustained open-beak criterion defined in Equation (1) at any point during the inspection, its value is set to 1 and remains unchanged for the remainder of the inspection. Therefore, CPR reflects the proportion of inspected individuals that exhibited at least one qualifying sustained open-beak episode during the inspection. The algorithm workflow is summarized in Algorithm 1.
**Algorithm 1:** CPR Estimation Pipeline
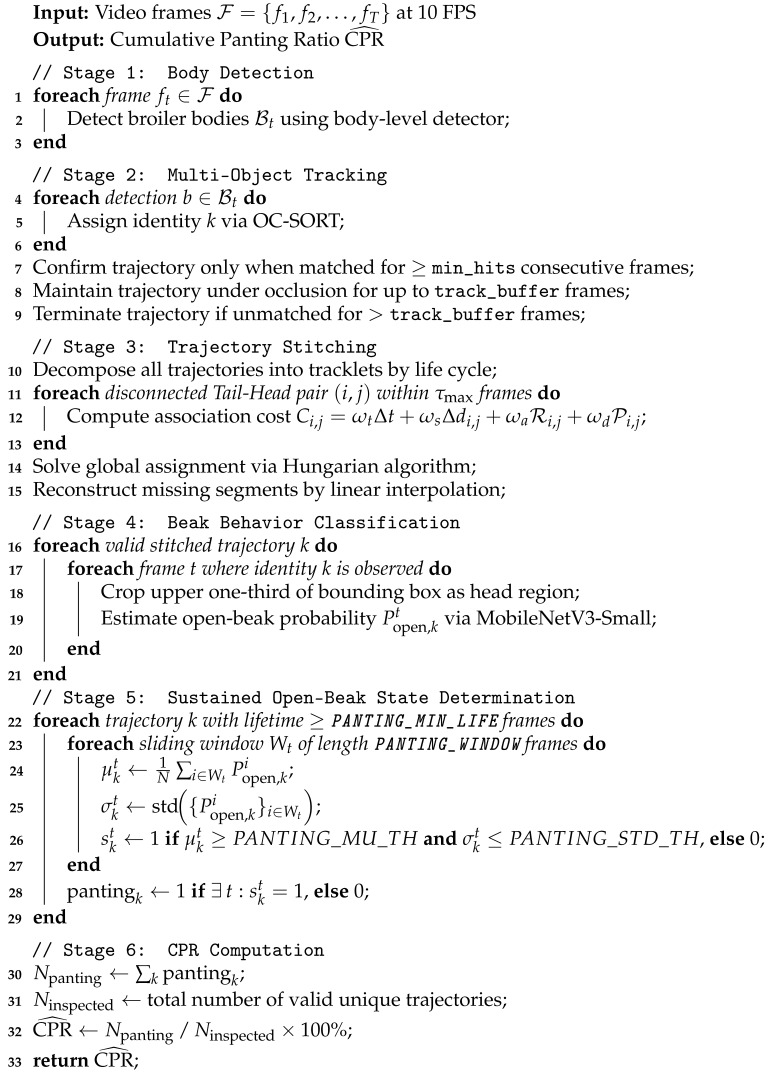


### 3.2. Cascaded Detection–Tracking–Classification Pipeline

To accurately estimate the proposed CPR during mobile inspection, the system must simultaneously accomplish two tightly coupled tasks: (1) continuously counting unique broiler breeders through MOT, and (2) identifying birds exhibiting sustained open-beak behavior through temporal open-beak behavior analysis. Therefore, the underlying task is not merely conventional MOT [30], but a behavior-aware tracking and quantification problem that requires both temporally stable identity association and fine-grained behavioral recognition.

To address this challenge, this paper proposes an improved framework based on OC-SORT [31], following the principle of “aggregating individual identities before inferring behavioral states.” OC-SORT introduces Observation-Centric Momentum and Observation-Centric Recovery mechanisms to improve trajectory recovery under occlusion and nonlinear motion conditions. Compared with conventional SORT-based trackers, it exhibits stronger robustness in fragmented tracking scenarios [32], making it particularly suitable for mobile inspection in multi-tier caged broiler breeder houses.

**Figure 6:**
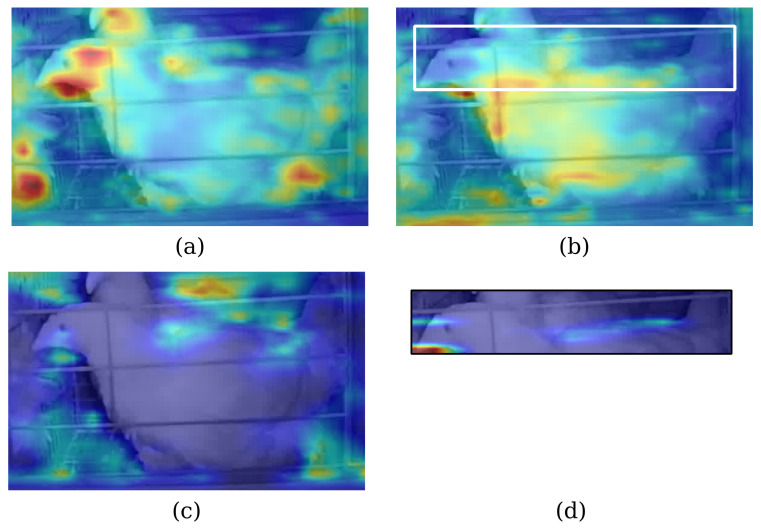
Grad-CAM comparisons of coupled and decoupled pipelines. For localization (top row), the coupled detector shows dispersed attention (**a**), whereas the decoupled detector focuses on body geometry (**b**). For classification (bottom row), the coupled detector attends to multiple body regions (**c**), while the decoupled MobileNet classifier focuses on the beak region (**d**).

However, directly integrating body-level tracking and beak-level behavior detection within a unified framework introduces substantial task conflicts. Reliable MOT-based counting requires temporally smooth and spatially stable body localization, whereas panting recognition depends on fine-grained local features around the beak region. During joint optimization, these competing objectives tend to interfere with each other, resulting in unstable bounding-box regression, as evidenced in Figure 6. Such instability further propagates into the downstream tracking stage, leading to trajectory fragmentation, object missing, and frequent ID switching, ultimately degrading counting performance and CPR estimation accuracy.

To address this issue, the original OC-SORT framework was reorganized into a cascaded architecture called the Cascaded Detection–Tracking–Classification pipeline, as illustrated in Figure 5. Instead of jointly optimizing tracking and behavior recognition within a single detector, the proposed framework physically decouples localization and behavioral inference into sequential stages. In the first stage, an object detector was employed and trained using dataset D1 as described in Section 2 exclusively for broiler breeder localization, aiming to generate temporally stable bounding boxes for downstream tracking. It is worth noting that while dataset D1 was originally annotated with two behavioral categories (open_beak and closed_beak), all annotated targets were merged into a unified broiler category using label collapsing. This strategy allows the detector to focus solely on body-level localization consistency rather than fine-grained behavioral discrimination.

The detector outputs were subsequently forwarded into the OC-SORT framework for identity assignment. After temporally stable tracking IDs had been established, a cascaded lightweight classifier based on MobileNetV3-Small [33] was introduced for open-beak recognition.

Notably, the training dataset for MobileNetV3-Small was constructed separately by reusing the original dataset D1. Instead of collecting additional annotations, spatial prior knowledge of bird anatomy was exploited, where the head region is typically located within the upper one-third of the body bounding box. Based on this prior, a heuristic spatial cropping strategy was applied to automatically extract head regions from the body bounding boxes generated by the upstream object detector, thereby reducing redundant background information and decreasing computational overhead. The cropped head regions were further resized to 64 × 64 pixels. With the estimated open-beak probability $P_{open}$ for each tracked ID *k*, sustained open-beak behavior can be identified following the definition introduced in Equation (1). Consequently, the proposed CPR can be computed as formulated in Equation (2).

### 3.3. Near-Online Trajectory Stitcher

**Figure 7:**
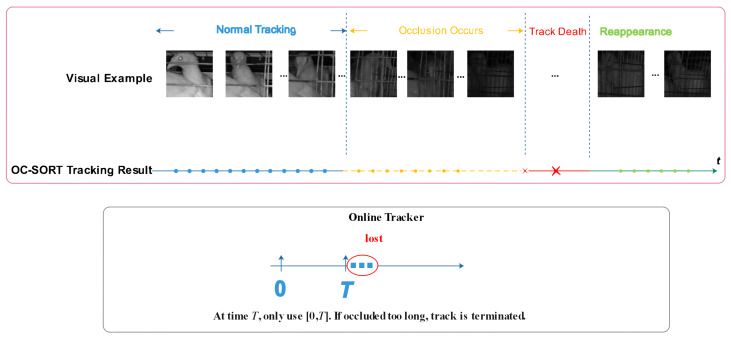
Over-counting reason in OC-SORT under occlusion conditions.

In caged rearing environments combined with a mobile inspection perspective, severe occlusions caused by cage wires and frequent inter-bird overlap are pervasive and unavoidable. Under such conditions, temporary target disappearance often prevents the detector from generating valid observations. Although OC-SORT employs Kalman filtering for short-term motion extrapolation, trajectories are still prematurely terminated once the occlusion duration exceeds the predefined track age threshold. Consequently, reappearing birds are frequently assigned new identities, leading to counting errors and compromised reliability in CPR estimation. This problem is illustrated in Figure 7.

**Figure 8:**
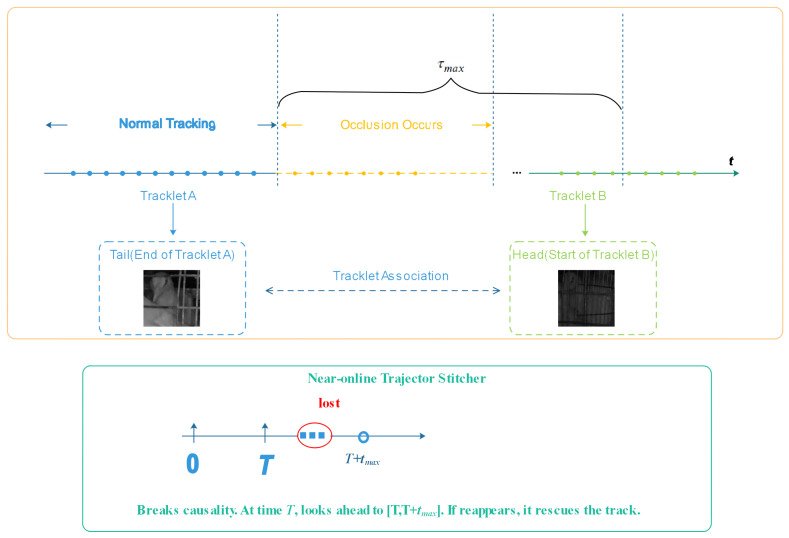
Workflow of the proposed Near-Online Trajectory Stitcher.

**Figure 9:**
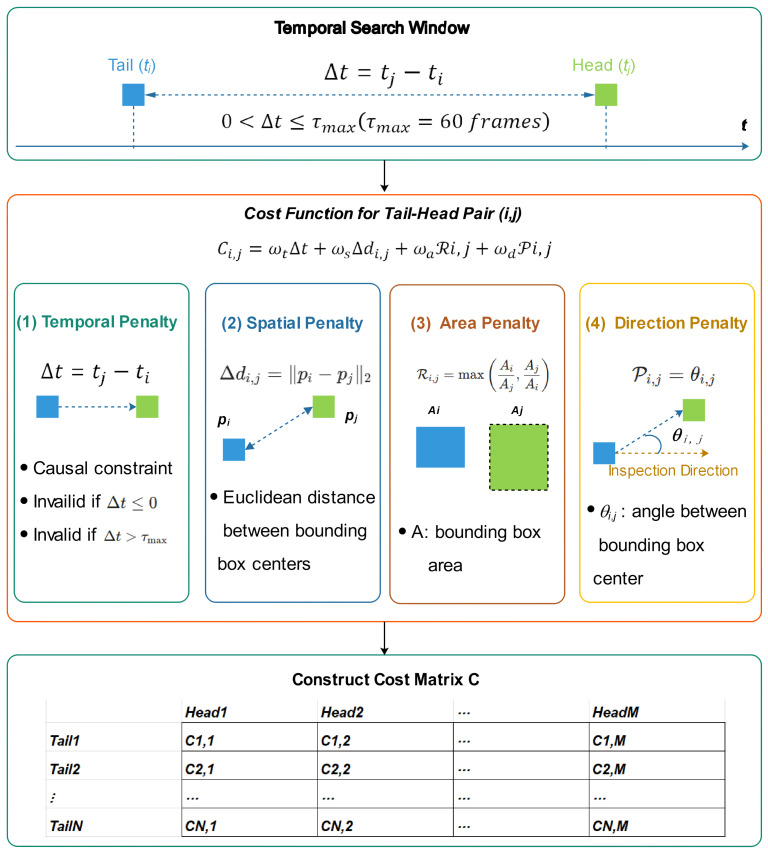
Construction of the trajectory association cost matrix.

To mitigate this issue, a Trajectory Stitcher module is proposed to repair fragmented trajectories under mobile inspection conditions. The proposed stitching framework consists of three sequential stages: tracklet extraction, trajectory association, and trajectory reconstruction. As illustrated in Figure 8, in the first stage, fragmented trajectories generated by the improved OC-SORT framework are decomposed into independent tracklets according to their life cycles. For each tracklet, the terminal frame (Tail) and the initial frame (Head) are extracted as candidate association endpoints. Since the mobile inspection platform follows a unidirectional scanning trajectory, birds permanently leaving the camera field of view are naturally treated as terminated trajectories rather than candidate association pairs. Subsequently, for any disconnected Tail–Head pair within the valid temporal search window $\tau_{max}$, a trajectory association cost matrix is defined to determine whether the two tracklets belong to the same broiler breeder. Each entry of the cost matrix is calculated as follows:

(3) $$C_{i,j}=\omega_{t}\Delta t+\omega_{s}\Delta d_{i,j}+\omega_{a}R_{i,j}+\omega_{d}P_{i,j}$$

where $\omega_{t},\;\omega_{s},\;\omega_{a},$ and $\omega_{d}$ denote the empirically determined hyperparameters for the corresponding penalty terms. The temporal penalty Δt constrains causal consistency and limits the association range. Associations are invalidated if Δt≤0 or exceeds the predefined threshold $\tau_{max}$. The spatial penalty $\Delta d_{i,j}$ evaluates the Euclidean distance between the bounding-box centers of candidate tracklets, while an adaptive search radius proportional to the bounding-box width is further enforced to suppress unrealistic spatial jumps. The area penalty $R_{i,j}$ measures bounding-box scale consistency to avoid mismatched identity associations caused by drastic morphological variations. Most importantly, the directional penalty $P_{i,j}$ explicitly incorporates the physical motion prior of the mobile inspection platform. As the camera moves with the inspection platform in an approximately fixed horizontal direction, valid trajectory associations are expected to follow the resulting global motion pattern in the image plane. Candidate associations contradicting the expected motion direction are therefore heavily penalized, effectively reducing identity switches caused by severe occlusion. This process is depicted in Figure 9.

**Figure 10:**
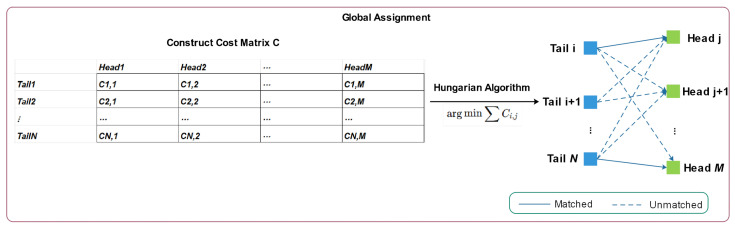
Trajectory reconnection by globally minimizing the association cost.

In the final stage, as shown in Figure 10, tracklet association is formulated as a linear assignment problem and solved globally using the Hungarian algorithm. Once a valid Tail–Head association is established, linear interpolation is applied to reconstruct the missing trajectory segment frame-by-frame across the occlusion interval, thereby generating temporally continuous and spatially smooth trajectories.

It should be noted that the proposed Trajectory Stitcher operates in a near-online manner rather than a strictly real-time fashion. By setting a maximum temporal search window of $\tau_{max}=60$ frames, the framework is essentially utilizing future observations to repair past trajectory fragmentation. Although this mechanism introduces an inherent latency of approximately 6 s at 10 FPS, such a delay is entirely acceptable for mobile poultry-house inspection, where heat stress evolves gradually rather than instantaneously. Therefore, sacrificing a marginal delay in exchange for improved trajectory continuity and reduced identity switching represents a practical trade-off for reliable CPR estimation.

### 3.4. Multi-Tier Parallel Sensing and Deployment Optimization

In mobile inspection scenarios, edge devices are constrained by limited computational resources while being required to process multiple video streams with high resolution and frame rate. In addition, tasks such as autonomous navigation and path planning are executed concurrently, further intensifying the competition for computational resource. This reveals a fundamental conflict between multi-stream perceptual demand and limited edge-side computational capacity, which is often overlooked in existing studies that primarily focus on model-level accuracy or latency optimization.

To address this issue, this paper introduces a multi-tier parallel sensing and deployment optimization framework. The core idea is to explicitly exploit spatial and temporal redundancies inherent in caged rearing environments, thereby improving computational efficiency without compromising perceptual performance.

Specifically, we first exploit the structural regularity of caged systems, where informative regions are consistently concentrated in the central band of each frame as shown in Figure 3. Based on this prior, redundant background regions are removed through ROI cropping, and the remaining regions are resized to a compact resolution for inference, reducing unnecessary spatial computation. Given the infrequency of behavioral transitions, inference is performed at a reduced frame rate. Frames from multiple streams are synchronized and aggregated into unified batches, enabling shared forward propagation and reducing redundant per-stream computation.

## 4. Results

This section presents a comprehensive evaluation of the proposed framework. After describing the experimental settings and implementation details, we compare the proposed method with state-of-the-art MOT approaches in terms of trajectory continuity and counting accuracy. Ablation studies are then conducted to examine the contribution of each proposed component. The system’s temporal stability is evaluated using a continuous 6 min video sequence, and its detector-agnostic capability is assessed by replacing the default detector with YOLO11s while keeping the remaining architecture unchanged. Finally, deployment performance and energy efficiency are evaluated on an edge-computing platform to assess the feasibility of the system for mobile inspection in multi-tier poultry houses.

### 4.1. Experimental Setting

As described in Table 1, dataset D1 was used to train and evaluate the object detector and open-beak classifier. D2 was used for the comparative evaluation, ablation studies, detector-agnostic analysis, and edge-deployment experiments presented in Section 4.3, Section 4.4, Section 4.6 and Section 4.7, respectively. D3 was used to evaluate the system’s temporal stability over an extended inspection period in Section 4.5.

The hyperparameters governing the proposed framework and their selected values are summarized in Table 2. To validate the rationality of the selected hyperparameters, a one-factor-at-a-time sensitivity analysis was conducted on five parameters that directly influence CPR estimation and trajectory association: PANTING_WINDOW, PANTING_MU_TH, PANTING_STD_TH, $\tau_{max}$ and $\omega_{a}$. Full results are provided in the Supplementary File, Tables S1–S3.

Except for edge deployment experiments, all other experiments were conducted on Ubuntu 18.04 using Python 3.9.0 and PyTorch 1.12.1, with CUDA 11.3 and CuDNN 8.3.2 for GPU acceleration. The hardware platform consisted of a 16-core Intel Core i9-11900K CPU and an NVIDIA GeForce RTX 3060 Ti GPU with 8 GB VRAM. YOLOX [34], which was natively integrated with OC-SORT, was adopted as the default detector. The hyperparameters of YOLOX and MobileNetV3-Small are shown in Table 3.

### 4.2. Evaluating CPR Under Commercial Conditions

To quantify the thermal load in each observation zone, the THI was first calculated based on the recorded temperature (*T*, in °C) and relative humidity (RH, in %) in dataset D4 using the following formula:

(4) $$\mathrm{THI}=T-\left(0.31-0.31\;\frac{RH}{100}\right)\;\left(T-14.4\right).$$

**Figure 11:**
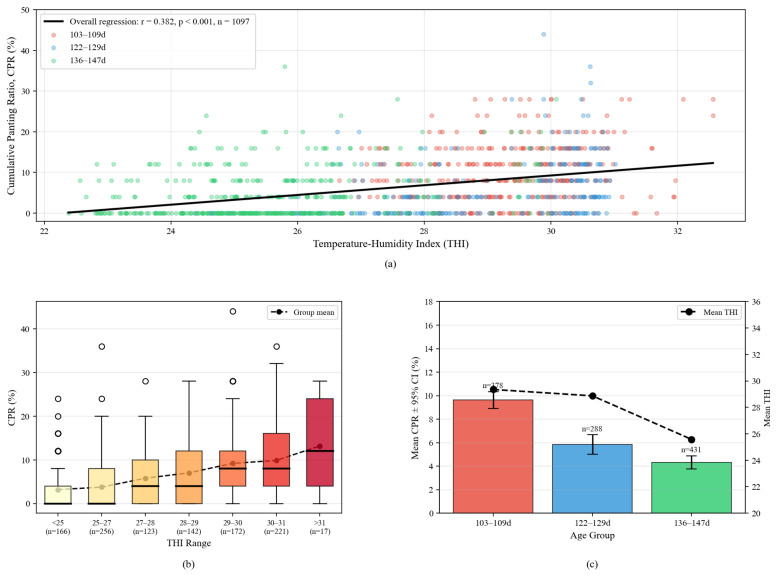
Practical validation of the CPR indicator. (**a**) Scatter plot of CPR against THI. (**b**) Box plots of CPR distribution across THI ranges, with group means connected by a dashed line. (**c**) Mean CPR (bars, ±95% CI) and mean THI (dashed line) across three observation periods (103–109, 122–129, and 136–147 days of age).

We then analyzed the correlation between CPR and THI. Wind speed and bird age were additionally included as covariates to account for environmental and biological factors. Results are shown in Figure 11.

Across the 1097 paired observations in dataset D4, CPR showed a positive correlation with the local THI (Pearson’s r=0.382, Spearman’s ρ=0.407, both p<0.001) and an inverse correlation with local wind speed (r=−0.156, p<0.001). As illustrated in Figure 11a, this overall positive THI–CPR relationship persisted across the monitored growth stages. A subsequent THI-stratified analysis (Figure 11b) demonstrated a monotonically increasing trend in CPR, with the group mean rising from 3.18% under mild conditions (THI<25) to 13.18% under severe thermal load (THI>31). A marked increase in both the mean and variance of CPR was observed at THI values of approximately 27–28. Furthermore, age-stratified comparisons (Figure 11c) indicated that variations in mean CPR across the three growth periods (103–109 d, 122–129 d, and 136–147 d) largely mirrored the underlying fluctuations in the ambient mean THI.

To disentangle the relative contributions of these co-varying environmental factors, a multivariate linear regression incorporating THI, wind speed, and bird age (*D*, in days) was constructed:

(5) $$\hat{\mathrm{CPR}}=1.325\;\mathrm{THI}-14.711\;v_{\mathrm{wind}}-0.015\;D-24.061$$

where $\hat{\mathrm{CPR}}$ is the predicted panting ratio (%), THI is the local Temperature–Humidity Index, $v_{\mathrm{wind}}$ is the local wind speed (m s^−1^), and *D* is bird age (days). The model achieved $R^{2}\;=\;0.212$ (F(3,1093)=97.90, p<0.001). Within this model, both THI (β=1.325, p<0.001) and wind speed (β=−14.71, p<0.001) served as significant independent predictors. Bird age did not emerge as a significant independent predictor within this observation window (β=−0.015, p=0.399). While the correlation magnitudes are moderate, they reflect the multifactorial nature of heat stress responses under commercial conditions, in which localized airflow, stocking microclimates, and individual variability exert effects beyond what THI alone captures. The independent contribution of wind speed—a variable absent from the standard THI formulation—indicates that CPR integrates environmental information that single-metric thermal indices do not represent. Taken together, these results suggest that CPR is sensitive to environmental heat load and may serve as a practical behavioral proxy for monitoring flock thermal status under commercial conditions.

### 4.3. Comparison with State-of-the-Art Tracking Frameworks Under Mobile Inspection Scenarios

To evaluate the proposed pipeline, we first compared it with several state-of-the-art tracking frameworks in terms of tracking performance and computational complexity. The evaluation metrics included Tracking Precision, Tracking Recall (%), MOTA (%), IDF1 (%), IDs, and Counting MAE for tracking accuracy, as well as the number of parameters (Params, M) and floating-point operations (GFLOPs) for model complexity assessment. Among these metrics, MOTA, IDF1, and IDs are standard evaluation indicators widely adopted in MOT tasks, while Params and GFLOPs are commonly used to measure model computational efficiency.

It should be noted that Tracking Precision and Tracking Recall in this study are evaluated based on the outputs of the tracking module rather than the raw detections generated by the object detector. Specifically, Tracking Precision is defined as

(6) $$P=\frac{\mathrm{TP}}{\mathrm{TP}+\mathrm{FP}}=\frac{\sum_{t}{\mathrm{TP}}_{t}}{\sum_{t}\left({\mathrm{TP}}_{t}+{\mathrm{FP}}_{t}\right)}$$

where ${\mathrm{TP}}_{t}$ denotes the number of successfully matched predictions in frame *t* under a predefined IoU threshold, and ${\mathrm{FP}}_{t}$ denotes the number of unmatched predicted boxes produced after temporal filtering and trajectory association. Tracking Recall is calculated as

(7) $$R=\frac{\mathrm{TP}}{\mathrm{TP}+\mathrm{FN}}=\frac{\sum_{t}{\mathrm{TP}}_{t}}{\sum_{t}{\mathrm{GT}}_{t}}$$

where ${\mathrm{GT}}_{t}$ represents the total number of ground-truth targets in frame *t*. To further evaluate counting consistency under mobile inspection scenarios, the Counting MAE metric was introduced:

(8) $$\mathrm{Counting}\;\mathrm{MAE}=\frac{1}{Num}\sum_{k=1}^{Num}\left|\hat{C_{k}}-C_{k}\right|$$

where Num denotes the total number of test videos, $C_{k}$ is the number of ground-truth unique identities in the *k*-th video, and ${\hat{C}}_{k}$ is the number of unique identities predicted by the tracker.

Unless otherwise specified, all trackers in the subsequent comparative experiments utilized YOLOX-S as the detector and were evaluated in a class-agnostic tracking setting (per_class = False). For ReID-based methods, OSNet_x0_25 was used as the appearance feature extractor, initialized with weights pretrained on MSMT17 and without further fine-tuning on livestock or poultry data. All tracking frameworks were implemented based on BoxMOT v17.0.0, except SORT, which used its original standalone implementation. Computational cost was reported as GFLOPs = MACs × 2. For ReID-based trackers, the computational cost of OSNet was estimated using the average number of detected targets per frame and included in the total cost. Deep OC-SORT [35] was excluded from the comparison because recurrent Cholesky decomposition failures caused a substantial number of frames to be skipped during inference. The tracking performance and computational complexity of different trackers are summarized in Table 4.

### 4.4. Ablation Study

Ablation experiments were conducted to investigate the contribution of each proposed method. All evaluation protocols and tracking metrics were kept fully consistent with the SOTA experiments.

The ablation results are summarized in Table 5, where Panting MAE is defined as follows:

(9) $$\mathrm{Panting}\;\mathrm{MAE}=\frac{1}{Num}\sum_{k=1}^{Num}\left|\hat{C_{kp}}-C_{kp}\right|$$

where Num denotes the total number of test videos, $C_{kp}$ is the number of ground-truth panting identities in the *k*-th video, and $\hat{C_{kp}}$ is the number of panting identities detected by the system. Config A represents the baseline OC-SORT framework with YOLOX-S as the default detector, without any additional modules. Config B introduces the proposed cascaded classification head on top of the baseline tracking pipeline. Config C integrates the proposed trajectory stitching algorithm into the baseline framework. Finally, Config D combines both the Trajectory Stitcher and the cascaded classification head, forming the complete proposed system. All configurations share identical detection and tracking settings to ensure a fair comparison of individual component contributions.

### 4.5. Accuracy and Temporal Stability of Automated CPR Estimation

To evaluate the agreement between system inference output and expert-labelled ground truth, the complete pipeline (Config D) was tested on both dataset D2 and D3. Table 6 presents the comparison across the three 1 min video clips. The system achieved a Counting MAE of 1.00, a Panting MAE of 1.33, and a CPR MAE of 10.00 percentage points. The mean bias (System-GT) was 1.00 birds, −1.33 birds, and −10.00 percentage points for inspected count, panting count, and CPR, respectively.

**Figure 12:**
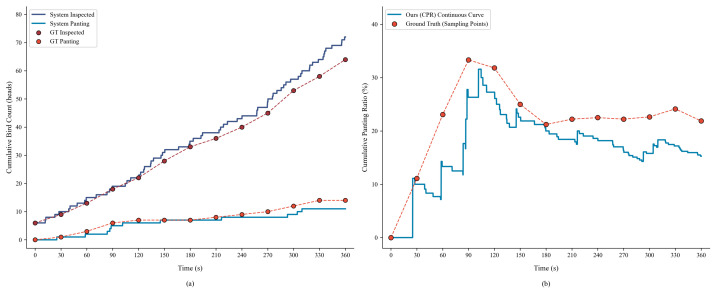
Comparison between expert observations and automated estimations during a 6 min continuous inspection. (**a**) Comparison of the total number of inspected birds and the number of panting birds. (**b**) Comparison of ground-truth CPR and automated CPR estimates.

Figure 12 compares the system CPR trajectory with expert observations at 30 s intervals over the 6 min continuous inspection. Across 13 valid observation points, the CPR MAE was 4.61 percentage points. Larger deviations occurred during the initial stage (30–90 s, CPR MAE: 5.97 percentage points), when the cumulative bird count was relatively small and individual counting errors had a larger impact. During the later stage (120–360 s), the CPR MAE decreased to 4.67 percentage points. The detailed per-observation comparison is provided in Table 7.

In both D2 and D3 evaluations, the estimated CPR showed a consistent negative bias relative to expert observations. This consistent conservative bias may be attributed to two practical factors during mobile inspection: additional birds from adjacent cage regions occasionally visible within the camera field of view increased the denominator, while missed panting individuals under occlusion and low illumination reduced the numerator. Nevertheless, the proposed framework maintained stable temporal CPR trends, indicating its potential for continuous flock-level quantification of sustained open-beak behavior associated with heat stress under practical inspection conditions.

### 4.6. Generalization Across Different Object Detectors

To further evaluate the robustness and generalization ability of the proposed framework, the default object detector was replaced from YOLOX-s to YOLO11-s, while keeping all other components of the pipeline unchanged. YOLO11 [41], released by Ultralytics in late 2024, introduces an improved backbone design and more efficient multi-scale feature fusion strategy compared to previous versions such as YOLOv8 and YOLOv10, achieving a better trade-off between detection accuracy and inference efficiency.

By substituting the detector with a newer architecture, we assess whether the proposed tracking and behavioral quantification pipeline remains stable under different detection backbones. The corresponding results are reported in Table 8.

### 4.7. Edge Deployment Performance and Energy Efficiency

After validating the tracking and behavioral quantification accuracy of the proposed framework, additional experiments were conducted on dataset D2 to evaluate its deployment performance on edge devices. These tests were specifically designed to simulate real-world, real-time inspection involving three concurrent video streams, where severe I/O and computational bottlenecks had previously been observed during multi-stream inference. This section compares the system performance and energy efficiency before and after deployment optimization.

The edge computing platform used in this study was an NVIDIA Jetson Orin NX 16G running Ubuntu 22.04, with JetPack 6.2, L4T 36.4.3, and TensorRT 10.3.0.30. The device operated under MAXN power mode, with the GPU frequency fixed at 918 MHz. The evaluation metrics included overall system throughput, energy efficiency, Counting MAE, and Panting MAE. The throughput and energy efficiency metrics were calculated as follows:

(10) $$T_{\mathrm{sys}}=\frac{N_{s}\times F}{t_{\mathrm{proc}}}$$

(11) $$\eta =\frac{T_{\mathrm{sys}}}{P_{\mathrm{avg}}}$$

where $T_{\mathrm{sys}}$ denotes the system throughput in FPS, $N_{s}$ is the number of video streams, *F* is the frame rate of each stream, $t_{\mathrm{proc}}$ is the average processing time in seconds, η denotes the energy efficiency in FPS/W, and $P_{\mathrm{avg}}$ is the average power consumption in Watts. In this study, $N_{s}=3$ and F=10 FPS.

Before optimization, the system employed the trained YOLO11s detector in FP32 precision using the original PyTorch (.pt) model, while the three video streams were processed sequentially in a polling manner. After optimization, the detector was converted into a TensorRT engine format (.engine) and executed in FP16 precision. In addition, multi-threaded concurrent I/O and cross-stream batch inference (Batch = 3) were introduced to jointly process the three video streams. In both settings, OC-SORT was adopted as the tracker, and MobileNetV3-Small served as the cascaded behavioral classification head.

**Figure 13:**
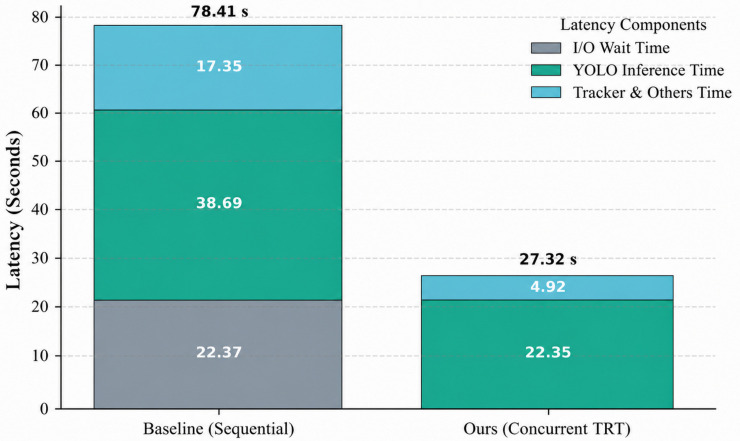
Latency breakdown comparing the sequential baseline and the proposed concurrent TensorRT pipeline on the Jetson Orin NX.

**Figure 14:**
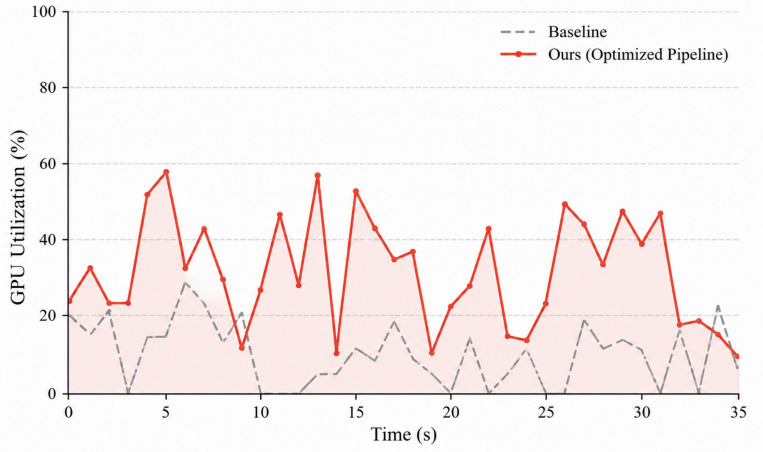
Comparison of GPU utilization during multi-stream perception execution.

**Figure 15:**
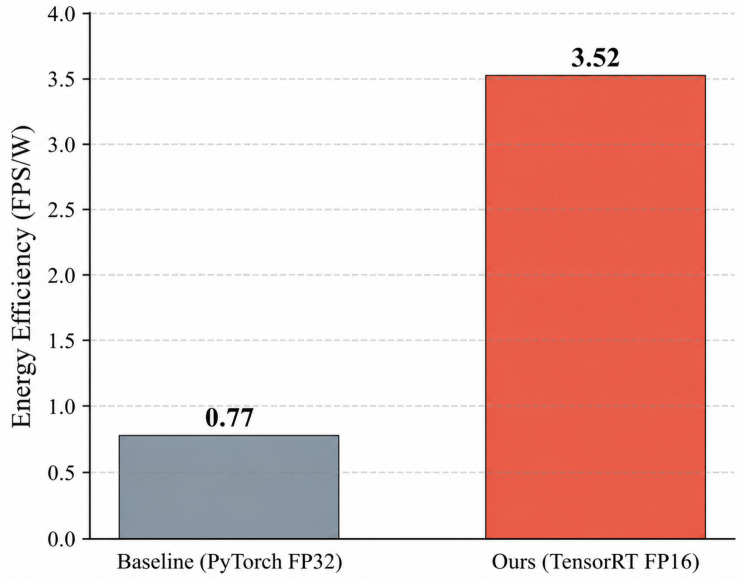
Comparison of system energy efficiency.

Experimental results showed that the proposed deployment optimization introduced no degradation in tracking or behavioral quantification accuracy. Specifically, the Counting MAE and Panting MAE remained unchanged at 1.67 and 1.33, respectively. Nevertheless, the optimized deployment strategy significantly improved the overall system energy efficiency and inference throughput. The detailed performance comparison is illustrated in Figure 13, Figure 14 and Figure 15.

## 5. Discussion

### 5.1. Frame-Level Estimation and Its Limitations

**Figure 16:**
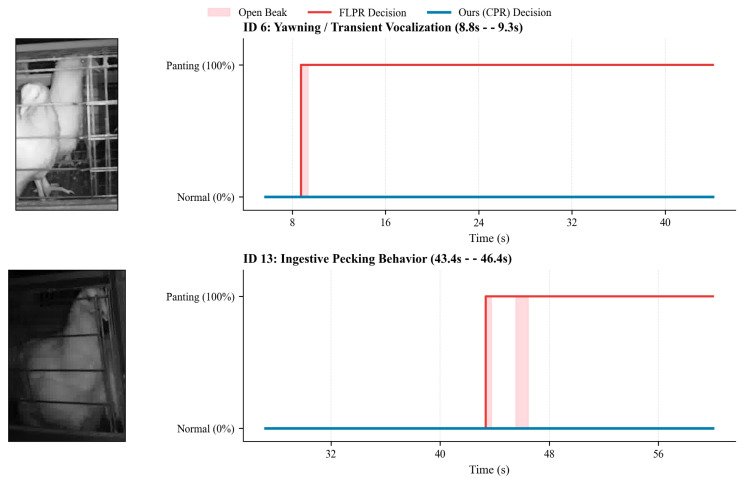
Comparison on typical negative examples. The CPR method effectively reduces the influence of transient behavioral variations, avoiding the false positives of the frame-level panting recognition (FLPR).

The ablation study results presented in Table 5 (Configs A and C) and the qualitative error cases in Figure 16 highlight the limitations of relying on instantaneous open-beak classification for flock-level quantification of sustained open-beak behavior associated with heat stress.

During housing inspections, broiler breeders display transient open-beak behaviors for non-stress activities, such as ingestive pecking or social vocalization. As illustrated in Figure 16:

- Transient Vocalization or Yawning (ID 6): During the tracking trajectory, ID 6 exhibited open-beak behavior for a single, brief interval of 0.6 s (6 frames) between 8.8 s and 9.3 s. The frame-level estimation baseline labeled this individual as “panting” immediately at the onset of the beak-opening and latched this state for the remainder of the observation.
- Ingestive Pecking Behavior (ID 13): ID 13 displayed a sequence characteristic of pecking at the feed trough, consisting of two short bursts of open-beak actions (0.4 s and 0.9 s) separated by a 2.0 s closed-beak interval. Instantaneous classification marked this individual as panting at 43.4 s.

These behaviors exhibit temporal patterns distinct from sustained panting, allowing them to be filtered out through temporal aggregation of open-beak predictions.

In addition to filtering transient open-beak events, CPR estimation requires identity-consistent tracking to accumulate behavioral evidence for each bird and prevent duplicate counting during mobile inspection.

### 5.2. Temporal Continuity for CPR Estimation

Since CPR is calculated as the proportion of individuals exhibiting sustained open-beak behavior among all uniquely inspected individuals, trajectory fragmentation and identity switches may introduce bias into both the numerator and denominator. As shown in Table 4, methods such as ByteTrack and BoT-SORT achieved strong overall tracking performance; nevertheless, tracking errors accumulated over extended inspection sequences and produced measurable counting deviations. This effect is captured by the Counting MAE, which directly reflects errors in the estimated number of uniquely inspected birds.

In contrast, the proposed framework achieved the lowest identity-switch count (IDs = 4) and the best Counting MAE despite not obtaining the highest MOTA score. This result indicates that identity preservation is particularly important for behavior-oriented mobile inspection, because tracking errors can accumulate into counting bias even when conventional frame-level tracking metrics remain competitive. In practical poultry-house environments, temporary occlusions caused by cage wires and dense bird clustering are inevitable. Once a trajectory is interrupted, a reappearing bird may be assigned a new identity and counted repeatedly, thereby introducing cumulative errors into CPR estimation.

Another notable observation is that ReID-based tracking methods did not consistently improve performance in the present scenario. Commercial broiler breeders in rearing cages exhibit a highly homogeneous appearance under low-illumination conditions, making it difficult for pretrained ReID models to extract sufficiently discriminative embeddings. Under severe occlusion, appearance-based association may therefore introduce additional identity ambiguity instead of improving temporal consistency.

### 5.3. Joint Effects of Decoupled Tracking and Trajectory Stitching

As shown in Table 5, ablation results demonstrate that accurate quantification of sustained open-beak behavior depends not only on tracking accuracy itself, but also on the temporal stability of behavioral observations. In the baseline configuration (Config A), open-beak detection and body-level tracking were jointly performed within the same detector. Although the tracker achieved relatively competitive MOTA and IDF1 scores, the Panting MAE remained extremely high (14.67), indicating that conventional MOT metrics alone cannot adequately reflect the reliability of long-term behavioral quantification.

After introducing the cascaded classification head (Config B), Panting MAE decreased substantially from 14.67 to 2.67, and IDs were also reduced from 10 to 7. In contrast, the other MOT metrics remained largely unchanged. This pattern is not contradictory. It reflects that tracking performance and behavioral quantification evaluate different components of the system. MOT metrics mainly assess bounding-box localization and identity association, which are determined by the upstream detector and tracker. Since Config B uses the same body detection backbone and tracking pipeline as Config A, its MOT performance is expected to remain similar.

The improvement in Panting MAE mainly results from more accurate temporal classification of individual open-beak behavior, which provides more reliable inputs for subsequent CPR aggregation. In Config A, bounding-box regression and beak-state classification share the same features. These coupled objectives weaken the features needed for fine-grained behavioral discrimination. This is consistent with the dispersed Grad-CAM activations shown in Figure 6. As a result, the per-frame probabilities are noisy. The sliding window cannot compensate for unstable behavioral observations, and therefore cannot ensure consistent CPR estimation. Config B separates localization from behavior classification. The body detector focuses on localization, while MobileNetV3-Small classifies cropped, resolution-preserved head regions. This design provides more stable open-beak probabilities. The sliding window can then accumulate behavioral evidence more accurately and suppress transient false positives. Therefore, Config B reduces Panting MAE without modifying the tracking pipeline.

The trajectory stitching module (Config C) further improved long-term identity continuity under severe cage-wire occlusions. Although its influence on Panting MAE was relatively limited, the stitching strategy significantly reduced counting errors and improved IDF1 performance by recovering fragmented trajectories across temporary observation gaps.

When both modules were jointly integrated (Config D), the framework achieved the best overall balance between trajectory continuity and behavioral quantification stability. Compared with the baseline configuration, the proposed system reduced Counting MAE from 3.00 to 1.00 while maintaining low identity-switch frequency. These results indicate that stable CPR estimation in mobile poultry inspection depends on both robust trajectory preservation and temporally consistent behavioral inference rather than solely optimizing frame-level tracking metrics.

### 5.4. Detector Robustness of the Proposed Quantification Pipeline

**Figure 17:**
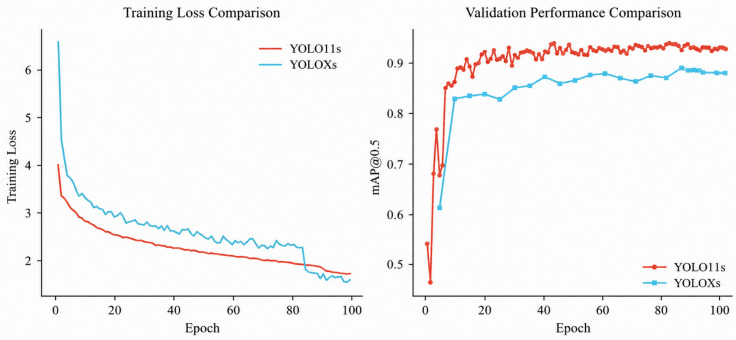
Comparison of mAP50 under different detectors.

In Section 4.6, YOLOX-S was replaced with the more recent YOLO11s while keeping all other components unchanged. During training, YOLO11s converged faster and achieved higher validation mAP@0.5 values than YOLOX-S, as illustrated in Figure 17. This indicates that YOLO11s provided stronger frame-level detection capability under the challenging low-light and occluded poultry-house conditions.

However, when integrated into the complete tracking and behavioral quantification pipeline, the performance gap between the two detectors became considerably smaller. As shown in Table 8, after introducing the proposed cascade classification and trajectory stitching modules, both detector configurations achieved highly comparable IDF1 and Counting MAE results. In particular, the complete YOLO11s-based configuration reduced the number of identity switches to only two while maintaining stable CPR-related quantification performance.

This observation suggests that reliable CPR estimation depends not only on detector accuracy, but more importantly on the preservation of temporal continuity throughout the tracking process. In practical mobile inspection scenarios, frame-level detection noise is unavoidable due to weak illumination, cage wire occlusion, and camera ego-motion. Although stronger detectors can provide more stable localization, the proposed cascade framework reduces the propagation of short-term detection errors into downstream behavioral statistics.

Another observation is that the proposed system achieves this robustness without relying on additional attention mechanisms or heavily customized detector structures. Instead, the improvement mainly originates from preserving temporal consistency and redesigning the interaction between detection, tracking, and behavioral inference. This characteristic makes the framework more adaptable to future detector upgrades and practical deployment in real mobile poultry inspection systems.

### 5.5. Edge Deployment Under Multi-Stream Mobile Inspection

Although the proposed framework achieved reliable tracking and CPR estimation performance, practical deployment in mobile poultry inspection still faces substantial computational constraints. In real multi-tier rearing environments, the inspection platform must simultaneously process multiple high-resolution video streams while maintaining timely responsiveness on resource-limited edge hardware. Under such conditions, the primary bottleneck is often no longer the detector itself, but the imbalance between video input throughput and available inference resources.

In the baseline implementation, the three video streams were processed sequentially using synchronous frame loading and FP32 inference. Although the detector and tracker remained functionally correct, this pipeline resulted in low hardware utilization and unstable real-time performance on the Jetson Orin NX platform. A major portion of execution time was consumed by frame decoding, memory transfer, and waiting overhead between consecutive inference stages, leading to insufficient GPU occupancy during mobile inspection (Figure 14).

To improve deployment efficiency, the proposed framework introduced asynchronous multi-stream processing together with TensorRT FP16 acceleration. By decoupling frame acquisition from YOLO inference and jointly batching frames from multiple cage layers, the system substantially reduced idle waiting time during deployment. Experimental results showed that the optimized pipeline increased throughput from 7.89 FPS to 51.43 FPS while reducing the total active processing time from 78.41 s to 27.32 s (Figure 13).

More importantly, the optimization improved practical energy efficiency rather than merely increasing raw computational throughput. Although the average power consumption increased moderately during high-load execution, the overall FPS/Watt ratio improved significantly because more inspection data could be processed within the same operating period (Figure 15). This characteristic is particularly important for battery-powered mobile inspection robots operating in large-scale poultry houses.

### 5.6. Limitations and Future Perspectives

Although the proposed framework improves the robustness of CPR-based sustained open-beak behavior quantification by integrating individual identity tracking with temporal aggregation, several limitations remain. First, some parameters in the proposed framework, including trajectory association thresholds and the temporal aggregation settings for panting assessment, were predefined based on domain knowledge and the characteristics of the inspection scenario. Although sensitivity analyses were conducted for representative parameters, more systematic optimization strategies under diverse conditions are required to further improve the adaptability of the framework. Second, although the validation datasets included birds of different ages and housing layers, all data were collected from the same commercial farm and flock. Further validation across multiple farms, breeds and environmental conditions is required to evaluate the generalization capability of the proposed method. Third, sustained open-beak behavior as detected by the proposed system is a behavioral proxy for panting rather than a direct physiological measurement. Although CPR showed a consistent association with environmental heat load indicators, additional physiological validation experiments would strengthen the interpretation of CPR as a quantitative behavioral indicator related to heat stress. Fourth, the current evaluation showed a consistent conservative bias in CPR estimation, mainly caused by occasional missed individuals exhibiting sustained open-beak behavior and additional visible birds from adjacent areas. Future studies will investigate calibration strategies to further reduce this bias.

## 6. Conclusions

This study presents a mobile inspection framework for flock-level quantification of sustained open-beak behavior in multi-tier caged broiler breeder houses. By integrating individual bird tracking with temporal open-beak behavior analysis, the proposed system enables continuous behavioral monitoring under commercial farming conditions. The resulting Cumulative Panting Ratio indicators showed associations with environmental heat load, suggesting their potential value for supporting heat stress-related management in precision poultry production.

The experimental results also indicate that temporal continuity is important for reliable behavioral quantification during mobile inspection. The proposed cascaded framework separates body-level tracking from head-level behavior classification, thereby improving tracking stability under low-illumination and cage-wire occlusion conditions. In addition, the trajectory stitching strategy integrates temporal, spatial, scale, and directional motion constraints to reconnect fragmented tracklets. Its consistent performance across different detector backbones further suggests that the framework is not strongly dependent on a specific detection architecture. Beyond the algorithmic contributions, this study highlights the practical importance of deployment-oriented optimization for multi-stream mobile inspection in commercial poultry houses.

Future work will migrate the edge-side implementation from Python to C++ in order to explore partial DLA offloading for compatible computational layers, enabling heterogeneous execution on the Jetson Orin NX. Onboard environmental sensors will be integrated with behavioral observations to support multimodal heat stress assessment in precision poultry management. Furthermore, controlled physiological and biochemical experiments will be conducted to examine the relationships between CPR and established heat stress measures, such as body temperature, respiratory rate, and relevant blood biomarkers, thereby providing stronger biological validation of CPR.

## Figures and Tables

**Table 1:** Summary of the four datasets used in this study.

Dataset	Purpose	Time	Scale	Annotation
D1	Detector and classifier training and validation	11 August 2025 26 August 2025 27 August 2025 28 August 2025	2000 train images 1260 val images	Bounding boxes + beak behavior class
D2	SOTA benchmark and ablation studies	31 August 2025 08:00 and 15:00 2 September 2025 20:00	3 clips × 60 s (1800 frames)	Frame-level identity + beak behavior class
D3	Long-sequence robustness evaluation	16 September 2025	1 clip × 360 s (3602 frames)	Expert observation and count at 30 s intervals
D4	Association between CPR and environmental heat load	1 August 2025– 25 September 2025	1097 observation records	Manual panting count + microclimate log

**Table 2:** Hyperparameter settings for the proposed CPR estimation framework.

Component	Parameter	Symbol	Value	Description
Tracking	Track initiation	min_hits	3 frames	Minimum consecutive matches to confirm a trajectory
	Occlusion buffer	track_buffer	30 frames	Maximum tolerated consecutive missed detections
Trajectory Stitcher	Stitching window	$\tau_{\max}$	60 frames	Maximum temporal gap for tracklet association
	Temporal weight	$\omega_{t}$	1.0	Penalty weight for temporal gap Δt
	Spatial weight	$\omega_{s}$	0.5	Penalty weight for spatial displacement $\Delta d_{i,j}$
	Direction weight	$\omega_{d}$	1.0	Penalty weight for directional inconsistency $P_{i,j}$
	Area ratio weight	$\omega_{a}$	5.0	Penalty weight for area ratio $R_{i,j}$
Panting Assessment	Min track life	PANTING_MIN_LIFE	10 frames	Minimum trajectory length for behavioral statistics
	Panting window	PANTING_WINDOW	10 frames (1 s)	Sliding window length for sustained open-beak behavior inference
	Mean threshold	PANTING_MU_TH	0.5	Minimum mean open-beak probability within window
	Std threshold	PANTING_STD_TH	0.35	Maximum std of open-beak probability within window

**Table 3:** Hyperparameter configurations for YOLOX and MobileNetV3-Small training.

Hyperparameter	YOLOX	MobileNetV3-Small
Input Size	640×640	64×64
Number of Output Classes	1 (broiler)	2 (open_beak / closed_beak)
Pretrained Weight	yolox_s.pth	MobileNet_V3_Small_Weights.
		IMAGENET1K_V1
		(mobilenet_v3_small-047dcff4.pth)
Training Epochs	100	5
Batch Size	16	128
Optimizer	SGD + Momentum	Adam
Learning Rate	0.0025 with decay	0.001 without decay
Data Augmentation	Off-Line + Online	Online

**Table 4:** SOTA tracker benchmark.

Tracker	MOTA (%)	IDF1 (%)	IDs	P (%)	R (%)	Counting MAE	Params (M)	GFLOPs
SORT [36]	87.54	84.47	42	93.91	94.08	10.00	8.93	26.5
ByteTrack [37]	89.33	92.39	7	94.99	94.37	3.33	8.93	26.5
OC-SORT [31]	88.32	90.36	10	97.17	91.08	3.00	8.93	26.5
StrongSORT [38]	88.80	89.94	35	96.50	92.52	3.00	9.13	27.4
BoT-SORT [39]	90.09	91.09	6	95.68	94.41	2.00	9.13	27.5
HybridSORT [40]	88.00	74.49	9	93.68	94.47	5.33	9.13	27.5
Ours	87.67	92.79	4	95.96	91.57	1.00	10.45	26.5

**Table 5:** Ablation studies with the proposed methods.

Model	MOTA (%)	IDF1 (%)	IDs	P (%)	R (%)	Count MAE	Panting MAE	GFLOPs	Params (M)
[A] YOLOX-S + OC-SORT	88.32	90.36	10	97.17	91.08	3.00	14.67	26.5	8.93
[B] + Cascade Head	88.30	91.43	7	97.24	90.95	2.00	2.67	26.5	10.45
[C] + Stitcher	87.80	93.69	4	95.93	91.73	1.33	12.67	26.5	8.93
[D] + Cascade Head + Stitcher	87.67	92.79	4	95.96	91.57	1.00	1.33	26.5	10.45

**Table 6:** One-to-one comparison between system-produced and ground-truth CPR on D2 (Config D).

Session	Inspected Count		Panting Count		CPR (%)	
	GT	System	GT	System	GT	System
31 August 2025 08:00	25	25	2	1	8.0	4.0
31 August 2025 15:00	14	15	2	1	14.3	6.7
2 September 2025 20:00	13	15	5	3	38.4	20.0
MAE	1.00		1.33		10.00	
Mean Bias	1.00		−1.33		−10.00	

**Table 7:** Observation point comparison between system-produced and ground-truth CPR on D3 (Config D).

Time Point(s)	Inspected Count		Panting Count		CPR (%)	
	GT	System	GT	System	GT	System
0	6	6	0	0	0.0	0.0
30	9	10	1	1	11.1	10.0
60	13	15	3	2	23.1	13.3
90	18	19	6	5	33.3	26.3
120	22	22	7	6	31.8	27.3
150	28	31	7	7	25.0	22.6
180	33	35	7	7	21.2	20.0
210	36	38	8	7	22.2	18.4
240	40	43	9	8	22.5	18.6
270	45	49	10	8	22.2	16.3
300	53	57	12	9	22.6	15.8
330	58	64	14	11	24.1	17.2
360	64	72	14	11	21.9	15.3
MAE (per observation)	2.77		1.23		4.61	
Mean Bias (per observation)	2.77		−1.23		−4.61	

**Table 8:** Performance under different detectors.

Model	MOTA (%)	IDF1 (%)	IDs	P (%)	R (%)	Count MAE	GFLOPs	Params (M)
[A] YOLO11s + OC-SORT	84.45	85.92	16	97.84	86.52	3.33	21.6	9.43
[B] + Cascade Head	84.84	90.25	6	97.86	86.80	1.67	21.6	10.95
[C] + Stitcher	84.38	89.33	11	96.54	87.64	2.00	21.6	9.43
[D] + Cascade Head + Stitcher	85.51	92.32	2	97.52	87.77	1.67	21.6	10.95

## Data Availability

The original data presented in the study are openly available in [Broiler breeders] at https://doi.org/10.5281/zenodo.21451846.

## Supplementary Materials

The following supporting information can be downloaded at: https://www.mdpi.com/article/10.3390/ani16152408/s1, Table S1. Classification and rationale of hyperparameters used in the proposed framework. Table S2. Sensitivity analysis of key hyperparameters affecting panting assessment on dataset D3. Table S3. Sensitivity analysis of hyperparameters in Trajectory Stitcher on dataset D2.
