# Supplementary material for: Robust Tracking and Quantification of Open-Beak Behavior for Heat Stress Assessment in Multi-Tier Caged Rearing Broiler Breeders via Mobile Inspection
Source: Animals (Basel). 2026 Aug 4;16(15):2408. doi: 10.3390/ani16152408 (PMC13463861; doi:10.3390/ani16152408)
Supplement: Supplementary file 1 [file animals-16-02408-s001.zip › animals-4447427-supplementary.pdf]

Sensitivity analyses were conducted on the following five hyperparameters: sliding window length, behavioral probability thresholds, maximum stitching gap, and area penalty weight (the third categories in Table S1), using the strategy of one-factor-at-a-time. As the results shown in Tables S2 and S3, the selected values consistently correspond to the optimal or near-optimal performance across all five parameters, with deviations in either direction leading to measurable performance degradation, which validates the rationality of the chosen experimental configuration.

It should be noted that sensitivity analyses were conducted on different datasets according to the evaluation objectives of each module. The behavioral inference parameters were evaluated on D3, where expert observations were available for direct Panting MAE comparison. In contrast, trajectory stitching parameters were evaluated on D2, which provides frame-level identity annotations required for multi-object tracking evaluation.

Table S1. Classification and rationale of hyperparameters used in the proposed framework.

Category	Hyperparameters	Treatment strategy
Domain baseline standards	min_hits<br>track_buffer	Adopted from established MOT configurations and fixed for comparability
Biological prior constraints	PANTING_MIN_LIFE	Initialized according to the temporal persistence characteristics of panting behavior and locally examined
Sensitivity-analyzed parameters	PANTING_WINDOW<br>PANTING_MU_TH<br>PANTING_STD_TH<br>τ_{max}<br>ω_a<br>ω_t<br>ω_s<br>ω_d	Directly influence CPR estimation or trajectory association; evaluated through sensitivity experiments

Table S2. Sensitivity analysis of key hyperparameters affecting panting assessment on dataset D3.

Hyperparameter	Tested value	Panting MAE (birds/observation) ↓
PANTING_WINDOW	4 frames (0.4 s)	6.67
	6 frames (0.6 s)	4.27
	8 frames (0.8 s)	2.33
	10 frames (1.0 s) [used in this study]	1.23
	12 frames (1.2 s)	1.23
	15 frames (1.5 s)	1.21
	20 frames (2.0 s)	3.31
PANTING_MU_TH	0.35	2.67
	0.40	2.31
	0.45	1.40
	0.50 [used in this study]	1.23
	0.55	1.26
	0.60	4.72
	0.65	4.33
PANTING_STD_TH	0.15	3.67
	0.20	3.33
	0.25	2.00
	0.30	1.56
	0.35 [used in this study]	1.23
	0.40	1.78
	0.45	2.00

Table S3. Sensitivity analysis of hyperparameters in Trajectory Stitcher on dataset D2.

Hyperparameter	Tested value	IDs	IDF1 (%)	Count MAE (birds/video) ↓
τ_{max}	15 frames (1.5 s)	17	89.31	2.33
	30 frames (3.0 s)	10	90.43	1.67
	45 frames (4.5 s)	8	91.76	1.33
	60 frames (6.0 s) [used in this study]	4	92.97	1.00
	90 frames (9.0 s)	4	93.05	1.00
	120 frames (12.0 s)	3	93.62	1.00
ω_a	1.0	8	88.41	3.16
	2.5	7	90.83	3.00
	5.0 [used in this study]	4	92.97	1.00
	7.5	8	90.26	2.00
	10.0	11	88.73	2.11
	15.0	15	87.15	3.23
